# The Potential Functions and Beneficial Effects of Melatonin on Cognitive Impairment, Neuroinflammation, Blood–Brain Barrier Leakage, and Synaptic Dysfunction in the Offspring of Mice Exposed to Gestational Intermittent Hypoxia

**DOI:** 10.1002/brb3.71321

**Published:** 2026-03-31

**Authors:** Xue‐Yan Li, Yun‐Zhoug Cheng, Yue‐Ming Zhang, Fei Hu, Shi‐Kun Fang, Gui‐Hai Chen, Yu Wang

**Affiliations:** ^1^ Department of Neurology the First Affiliated Hospital of Anhui Medical University Hefei Anhui China; ^2^ Department of Neurology Anhui Public Health Clinical Center Hefei Anhui China; ^3^ Department of Neurology (Sleep Disorders) the Fourth Affiliated Hospital of Anhui Medical University Hefei Anhui China; ^4^ Department of Paediatrics the Fourth Affiliated Hospital of Anhui Medical University Hefei Anhui China

**Keywords:** cognitive function, intermittent hypoxia, melatonin, neuroinflammation, synaptic plasticity

## Abstract

**Introduction:**

Gestational intermittent hypoxia (GIH), which serves as a model for obstructive sleep apnea (OSA), is associated with adverse maternal and neonatal outcomes, especially cognitive impairments in offspring. Growing evidence supports that the anti‐inflammatory actions of melatonin significantly influence the peripartum environment and contribute to the mitigation of neurodegeneration. However, the full impact of GIH on offspring cognition and the molecular mechanisms by which melatonin modulates these effects remain uncertain. Thus, in this study, we explored the neurobiological changes in GIH‐exposed offspring and the mechanism underlying maternal melatonin supplementation in preventing these alterations using a murine model.

**Methods:**

C57BL/6J mice were exposed to GIH between gestational Days 15 and 21. Concurrently, dams received either vehicle or melatonin. The Morris water maze test was employed to evaluate offspring cognitive function, after which the offspring were euthanized at 2 months of age. The hippocampal levels of glial markers (ionized calcium‐binding adapter molecule 1 [Iba‐1], glial fibrillary acidic protein [GFAP]), NOD‐like receptor thermal protein domain‐associated protein 3 [NLRP3], nuclear factor‐kappa B [NF‐κB], tight‐junction proteins (zonula occludens‐1 [ZO‐1], occludin), and synaptic plasticity‐related proteins (brain‐derived neurotrophic factor [BDNF], tropomyosin receptor kinase B [TrkB], postsynaptic density protein 95 [PSD‐95], synaptophysin [SYN]) were quantified by enzyme‐linked immunosorbent assay and western blot.

**Results:**

Maternal melatonin supplementation significantly attenuated learning and memory impairments, reduced the protein levels of Iba‐1 and GFAP by suppressing NLRP3/NF‐κB signaling, and elevated those of ZO‐1, occludin, BDNF, TrkB, PSD‐95, and SYN. Additionally, melatonin mitigated inflammatory responses, glial cell activation, blood–brain barrier (BBB) leakage, and synaptic dysfunction induced by GIH in mice.

**Conclusions:**

Our results demonstrated that GIH‐exposed mice exhibit cognitive deficits, alongside neuroinflammatory responses, leading to inflammasome activation, glial reactivity, BBB breakdown, and synaptic deficits. However, melatonin exerted significant protective effects against these deleterious effects.

## Graphical abstract

Melatonin rescues the cognitive deficits induced by gestational intermittent hypoxia exposure in male offspring mice, and these results were associated with neuroinflammation, BBB integrity, and alterations in synaptic function.

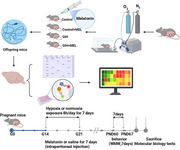

## 1 Introduction

Obstructive sleep apnea (OSA) is a prevalent sleep‐related breathing condition characterized by recurrent upper airway collapse. These episodes are accompanied by oxygen desaturation, hypercapnia, and sleep fragmentation, which collectively contribute to daytime somnolence, diminished concentration, hypertension, and various cardiovascular, metabolic, and cognitive impairments (Gottlieb et al. 2020). Recently, the rising prevalence of OSA among pregnant women has been linked to a heightened risk of perinatal diseases and neonatal complications (Bonsignore et al. 2019; Warland et al. 2018). Factors such as obesity, fluid retention, and altered lung mechanics—specifically reduced functional capacity and residual volumes—likely account for the growing incidence of OSA within this population (Warland et al. 2018). A recent meta‐analysis associated OSA with a 15% pooled prevalence during mid‐to‐late pregnancy and identified the disorder as a predictor of maternal hypertension, gestational diabetes, preterm delivery, and low Apgar scores (Liu et al. 2019). Evidence suggests that gestational intermittent hypoxia (GIH) resulting from maternal OSA is associated with increased placental weight and neonatal adiposity. These findings indicate that alterations in placental functionality may have long‐term health implications for infants, potentially extending into adulthood (Perkins and Einion 2019). Notably, recent studies have demonstrated that chronic intermittent hypoxia (CIH) can result in cognitive dysfunction in adolescent rodent offspring (Mabry et al. 2023; Vanderplow et al. 2022; Cristancho et al. 2022). However, the specific impact of GIH on cognitive decline in offspring remains to be elucidated, as do the underlying mechanisms.

Neuroinflammation and oxidative stress induced by intermittent hypoxia (IH) are considered the primary mechanisms underlying OSA‐related neurocognitive impairment (Sapin et al. 2015; Shiota et al. 2013). Recent studies have found that IH results in increased activity of hypoxia‐inducible factor‐1 alpha (HIF‐1α) in the context of OSA and its associated complications. Specifically, OSA‐related IH triggers peripheral inflammation that eventually affects the CNS either through the blood–brain barrier (BBB) disruption or via vagal afferent signaling (Roche et al. 2024). Furthermore, a series of studies has demonstrated that neuroinflammation in OSA plays a crucial role in the dysregulation of hippocampal synaptic plasticity and the progression of neurocognitive deficits (Yilmaz Avci et al. 2017; Liu et al. 2020).

High levels of CNS inflammation further upregulate the activity of microglia (ionized calcium‐binding adapter molecule 1 [Iba‐1^+^]) and astrocytes (glial fibrillary acidic protein [GFAP^+^]), thereby initiating and amplifying the neuroinflammatory response. Notably, IH can directly activate these primary glial populations, further exacerbating the inflammatory response in the CNS (Liu et al. 2020; Kiernan et al. 2016; Kiernan et al. 2019). Microglia and astrocytes are essential for CNS homeostasis, mediating developmental synaptic remodeling, providing neurotrophic support, facilitating neurotransmitter clearance, and maintaining BBB integrity. However, the mechanisms linking GIH to glial dysregulation remain unknown.

An exaggerated neuroinflammatory response could subsequently enhance glial cell activation and increase BBB permeability, leading to intracranial inflammation that culminates in synaptic dysfunction and neurocognitive deficits (Norden et al. 2016; Di Benedetto et al. 2022; Liu et al. 2018; Zhang et al. 2022). Studies have demonstrated that lipopolysaccharide (LPS)‐induced neuroinflammation can downregulate the levels of ZO‐1 and occludin, which are critical cytoplasmic tight‐junction proteins and established indicators of BBB integrity (Zou et al. 2022). Evidence strongly supports that BBB permeability increases in the context of IH and sleep fragmentation, accompanied by increases in the activities of nuclear factor‐kappa B (NF‐κB) and tumor necrosis factor‐alpha (TNF‐α) (Lim and Pack 2014; Puech et al. 2023; Voirin et al. 2023; Fei et al. 2021). Furthermore, research has shown that serum inflammatory biomarkers, including various interleukins, TNF‐α, and the NOD‐like receptor thermal protein domain‐associated protein 3 (NLRP3) inflammasome, are activated in animal models of OSA (Díaz‐García et al. 2022; Yangzhong et al. 2024). Specifically, CIH induces inflammation via the NF‐κB/NLRP3 signaling pathway, which triggers the secretion of large amounts of IL‐1β by microglia, leading to synaptic plasticity impairment and cognitive dysfunction (Mishra et al. 2021).

Synaptic proteins, including brain‐derived neurotrophic factor (BDNF), postsynaptic density protein 95 (PSD‐95), and synaptophysin (SYN), are key markers of synaptic plasticity. These proteins perform multiple functions within the nervous system, encompassing the regulation of neuronal development and the maintenance of long‐term synaptic plasticity in the hippocampus and other brain regions (De Vincenti et al. 2019; Dore et al. 2021; Liu et al. 2010). Evidence suggests that BDNF/tropomyosin receptor kinase B (TrkB) deprivation increases inflammatory cytokine levels and may serve as an important regulator of amyloidogenic processing (Costa et al. 2022). Furthermore, research has revealed a direct interaction between neuroinflammation and the levels of SYN and PSD‐95 during critical developmental periods for new hippocampal neurons (Chugh et al. 2013; Sheppard et al. 2019). We previously reported that maternal exposure to LPS during the late trimester of pregnancy reduces synaptic protein expression and impairs long‐term cognitive function in offspring. Similarly, neonatal IH significantly reduces BDNF and SYN expression in the ipsilateral medial prefrontal cortex (Tata et al. 2021). These findings indicate that neuroinflammation can drive the depletion of synaptic proteins and impair cognition following hypoxia or LPS exposure.

Melatonin is a pleiotropic molecule that acts as a potent scavenger of reactive oxygen and nitrogen species, exerts anti‐inflammatory effects, enhances immune responses, and modulates circadian rhythms (Sanchez‐Barcelo et al. 2017). Substantial evidence supports that the anti‐inflammatory activities of melatonin significantly influence the peripartum period and adult life (Chitimus et al. 2020). Patients with neurological disorders, such as Alzheimer's disease and mild cognitive impairment (MCI), typically exhibit reduced melatonin levels (Mishima et al. 1999; Obayashi et al. 2015). Furthermore, recent research has suggested that prolonged melatonin treatment reduces pro‐inflammatory cytokine levels in aged mice, highlighting its potential for averting age‐associated memory decline (Permpoonputtana et al. 2018). Given its rapid metabolism, the neuroprotective benefits of melatonin may be mediated by metabolites such as 6‐hydroxymelatonin, indolic, and kynuric metabolites (Slominski et al. June 2025 Jun; Kim et al. 2013). In view of its potential influence on aging and psychiatric disorders, as well as its abundant presence in several human tissue types, melatonin represents a promising alternative pharmacological treatment for neurodegenerative diseases. Given that late gestation is a critical window for neurodevelopment, this period constitutes an optimal time for initiating melatonin administration. Therefore, it is essential to investigate whether maternal melatonin supplementation initiated during late embryonic development protects offspring against the deleterious effects of GIH.

Thus, we hypothesized that, in the context of OSA, increased NF‐κB/NLRP3 pathway activity leads to the activation of microglia and astrocytes, which subsequently release pro‐inflammatory cytokines, thereby modulating neuroinflammatory responses. These effects may be associated with impaired synaptic plasticity and cognitive impairment. In the present study, we examined whether GIH exposure led to alterations in the hippocampal expression of neuroglial markers (Iba‐1 and GFAP), NF‐κB p65, NLRP3, tight‐junction proteins (ZO‐1 and occludin), and synaptic plasticity markers (BDNF/TrkB, PSD‐95, and SYN). Subsequently, we investigated whether maternal melatonin supplementation, initiated during gestation, provides a protective effect against GIH‐induced changes in these inflammatory and neuroplasticity markers.

## 2 Materials and Methods

### 2.1 Animals and Treatments

**FIGURE 1:**
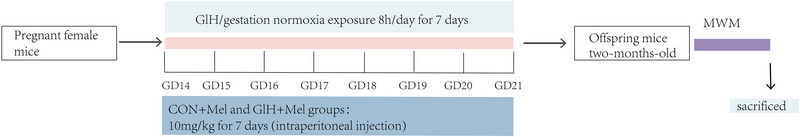
Animal experimental protocol. CON, control group; GD, gestational day; GIH, gestational intermittent hypoxia group; GIH+Mel, GIH plus melatonin group; Mel, melatonin group; MWM, Morris water maze test.

All animal‐related experimental procedures were approved by the Animal Ethics Committee of Anhui Medical University. C57BL/6J mice were purchased from Beijing Vital River Laboratory Animal Technology Co., Ltd, and were raised under conventional conditions until reaching 2 months of age. Following mating, the presence of a vaginal plug on the subsequent morning was used to identify Day 0 of gestation (GD 0), at which point females were housed individually. Subsequently, a model of GIH was used to mimic the most common clinical scenario of OSA. Pregnant mice were randomly divided into the following groups: control (CON), gestational intermittent hypoxia (GIH), melatonin only (Mel), and GIH with melatonin (GIH+Mel). After birth, pups were housed with their mothers until weaning on postnatal Day 21, after which they were categorized by maternal treatment regimen (*n* = 8 per group; CON, GIH, Mel, GIH+Mel). Behavioral and molecular analyses were conducted once male offspring reached 2 months of age (see Figure 1). All animal experiments were carried out following the ethical protocols established by the Center for Laboratory Animal Sciences and the Association of Laboratory Animal Sciences at Anhui Medical University (Approval No. LLSC20190710), ensuring humane care and use throughout the study.

### 2.2 GIH Treatment

In the GIH treatment groups, pregnant females were exposed to IH from GD 15 using the ProOx‐100 animal hypoxia control system. Treatment lasted for 7 consecutive days and was applied from 9 to 17 h daily. The oxygen concentration fluctuated between 21% and 10% within a 90‐s cycle. This protocol served to establish a model for late‐GIH (Wang et al. 2023). Females in the CON group were placed in the same environment but were exposed to normoxic air.

### 2.3 Drug Administration

Animals assigned to the Mel and GIH+Mel groups were administered melatonin intraperitoneally at 10 mg/kg once daily (purity ≥98%, product no. M5250 [powder], Sigma‐Aldrich) for a total of 7 days, as previously reported (Lee et al. 2019). This dosage (10 mg/kg) was used because it attains peak plasma melatonin concentrations of approximately 1 µM within 15 min in adult mice. Furthermore, a dose of 10 mg/kg has been demonstrated to provide neuroprotection in offspring exposed to gestational inflammation or hypoxia without compromising maternal or fetal safety. Animals in the CON and GIH groups were administered an equivalent volume of saline daily throughout the same period.

### 2.4 Morris Water Maze Test

Spatial learning and memory in adolescent male mice were assessed using the Morris water maze (MWM) over 5 consecutive days, with eight mice per group. The apparatus comprised a white circular pool (120 cm in diameter) partitioned into four identical quadrants and containing a submerged platform (10 cm in diameter, 24 cm in height). The water temperature was maintained at 21°C–23°C. Testing was conducted in a brightly illuminated white room, with distinct visual cues placed on the walls to provide spatial reference points for the mice. The experiment comprised two phases conducted over a 5‐day period: a learning phase (navigation task) and a memory phase (probe trial task). During the learning phase, mice underwent four training trials daily for 5 days, with the hidden platform remaining at a fixed location. Each trial started from a different quadrant. Mice were allowed to swim until they located the platform and then allowed to rest on it for 30 s. If a mouse failed to locate the platform within 60 s, it was gently guided to the location by the experimenter. In the memory phase, the platform was removed, and mice were released into the water from the quadrant opposite the target quadrant and were allowed to swim freely for 60 s. The following parameters were analyzed using ANY‐Maze software (Stoelting, USA): escape latency, distance swam to reach the platform, and swimming speed during the learning phase; and the percentage of distance swam and time spent in the target quadrant during the memory phase. All MWM tests were performed by an investigator blinded to the experimental condition; cage cards were color‐coded, and were decoded only after statistical analysis.

### 2.5 Real‐Time Fluorescence‐Based Quantitative PCR

Total RNA was reverse‐transcribed into cDNA using a commercial kit (TaKaRa, RR047A), and the resulting cDNA served as the template for quantitative PCR (qPCR). Each 10‐µL reaction contained 2× SYBR Green Mixture (5 µL), forward and reverse primers (1 µL each), cDNA (1 µL), and RNase‐free water (2 µL). The cycling conditions were as follows: initial denaturation at 95°C for 1 min, followed by 40 cycles of 95°C for 20 s and 60°C for 1 min. Relative mRNA abundance was determined via the 2^−ΔΔCt^ method. The sequences of the primers used are listed in Table 1.

**TABLE 1:** Sequences of the primers used for qPCR (5′–3′).

Gene	Amplicon size (bp)	Forward primer (5′–3′)	Reverse primer (5′–3′)
GAPDH	169	GCAGTGGCAAAGTGGAGATTG	CGCTCCTGGAAGATGGTGAT
NF‐κB p65	119	GCTCCTGTTCGAGTCTCCAT	TTGCGCTTCTCTTCAATCCG
NLRP3	176	GCTGCTATCTGGAGGAACTT	TGAGGTCCACATCTTCAAGG
GFAP	169	CTCCATAAAGGCCCTGACAT	GGAGTCATTCGAGACAAGGA
Iba‐1	103	AAAGTCAGCCAGTCCTCCTC	TTGCTAACTCCCAGGCATCA
Occludin	169	GTTTCAGGTGAATGGGTCAC	TCCCAAGATAAGCGAACCTG
ZO‐1	182	TTCCGGGGAAGTTACGTG	GGGACAAAAGTCCGGGAA
PSD‐95	110	GCTCCCTGGAGAATGTGCTA	TGAGAAGCACTCCGTGAACT
SYN	124	GCCTACCTTCTCCACCCTTT	GCACTACCAACGTCACAGAC
BDNF	94	TTACTCTCCTGGGTTCCTGA	ACGTCCACTTCTGTTTCCTT
TrkB	104	TCTGGAGGGTGCTATGCTAT	GGGGCAGAAACTCCAGAAAA

### 2.6 Western Blotting

Fifteen days after the completion of the MWM trial, the mice were euthanized by cervical dislocation. The brains were quickly removed, and the hippocampus was isolated and thoroughly homogenized. For total protein extraction, the homogenized tissue was incubated in RIPA lysis buffer. The resulting lysate was centrifuged at 12,000 rpm for 15 min, and the total protein was collected from the supernatant. Following protein quantification, the samples were mixed with 5× SDS‐PAGE loading buffer at a ratio of 1:4 and heated in boiling water for 10 min to ensure complete denaturation of the proteins. Equal amounts of protein from each sample were separated using SDS‐PAGE and then transferred to a PVDF membrane. Following blocking in Western Closure Solution for 2 h, the membranes were incubated with the following primary antibodies overnight at 4°C: anti‐Iba‐1 (1:500, SC‐32725), anti‐NF‐κB p65 (1:5000, ab16502), anti‐NLRP3 (1:1000, DF7438), anti‐GFAP (1:1000, ab279289), anti‐BDNF (1:1000, BA0565), anti‐occludin (1:1000, bs‐10011R), anti‐TrkB (1:1000, bs‐10011R), anti‐ PSD‐95 (1:2000, ab238135), anti‐SYN (1:1000, bs‐8845R), and anti‐GAPDH (1:2000, TA‐08). After washing, the membrane was probed with HRP‐linked goat anti‐rabbit/mouse IgG (1:20,000, ZB‐2301/ZB‐2305, ZSbio, USA) and visualized using an enhanced chemiluminescence reagent kit (340958, Thermo). The results of the western blot were analyzed using ImageJ software, with protein expression levels quantified by comparing the relative intensities of the protein bands.

### 2.7 Statistical Analysis

All statistical analyses were performed using GraphPad Prism 8.0. For the MWM test, repeated‐measures analysis of variance (ANOVA) was applied to evaluate escape latency and swimming distance data, followed by Tukey's post‐hoc tests for multiple comparisons. To assess data from other experimental procedures, including the memory phase of the MWM test, western blotting, and RT‐PCR, one‐way ANOVA was used. All data are presented as means ± standard error of the mean (SEM), and a *p* value of < 0.05 was considered statistically significant.

## 3 Results

### 3.1 Melatonin Alleviated GIH‐Induced Learning and Memory Dysfunction

**FIGURE 2:**
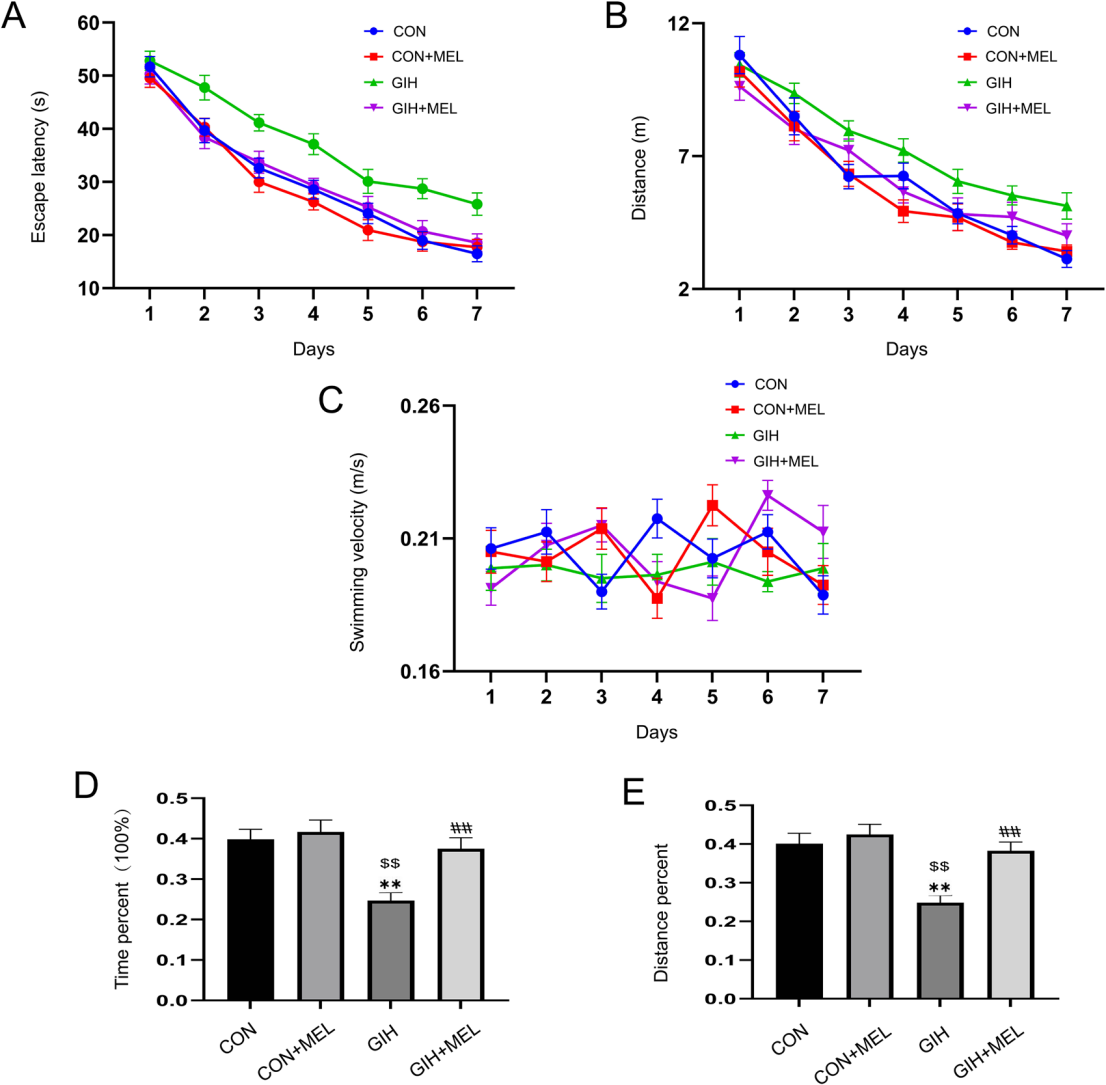
In the Morris water maze (MWM) test, melatonin exerted significant protective effects against gestational intermittent hypoxia (GIH)‐induced learning and memory deficits. In the learning phase, three key parameters were assessed across the four groups: (A) escape latency, (B) distance swam, and (C) average swimming speed. During the memory phase, the four groups were compared regarding (D) the percentage of time spent in the target quadrant and (E) the percentage of distance swam in the target quadrant. The escape latency, distance swam, and swimming velocity were analyzed by repeated measures ANOVA. The percentage of time spent and distance swam in the target quadrant were analyzed by one‐way ANOVA with Tukey's post hoc test; *p < 0.05, **p < 0.01 versus the CON, ^$^ *p* < 0.05, ^$$^ *p* < 0.01 versus the CON+Mel, ^#^ *p* < 0.05, ^##^ *p* < 0.01 versus the GIH.

The MWM test was conducted to evaluate cognitive function in mice. In the learning trials, the escape latency and distance swam were significantly different among groups (*F*_(3, 28)_ = 34.07, *p* < 0.01; *F*_(3, 28)_ = 11.7, *p* < 0.01) and across training days (*F*_(6, 168)_ = 143.60, *p* < 0.01; *F*_(6, 168)_ = 92.65, *p* < 0.01) (Figures 2A,B). There was no interaction effect of group × training day (*F*_(18, 168)_ = 0.89, *p* = 0.59; *F*_(18, 168)_ = 0.73, *p* = 0.78) (Figures 2A,B). Mice in the GIH group exhibited significantly longer escape latencies and distances swam in the learning trials than mice in the CON and Mel groups (all *p* < 0.05; Figures 2A,B). Notably, melatonin administration mitigated the effects of GIH, as evidenced by the reduced escape latency and distance swam in the GIH+Mel group relative to the GIH group (*p < *0.05; Figures 2A,B). However, no significant differences in swimming velocity were observed among the four groups (*p* > 0.05; Figure 2C). During the probe trial, one‐way ANOVA revealed significant inter‐group differences in the percentage of time spent in the target quadrant (*F*_(3, 28)_ = 9.174, *p < *0.05) and the percentage of distance swam in the target quadrant (*F*_(3, 28)_ = 7.80, *p < *0.05) (Figures 2D,E). Mice exposed to GIH exhibited memory impairment, characterized by significantly lower percentages of time spent and distance swam in the target quadrant compared with controls (all *p < *0.05; Figures 2D,E). In contrast, melatonin administration ameliorated these cognitive deficits in GIH mice, as indicated by increases in both the time spent and distance swam in the target quadrant relative to the GIH group (all *p < *0.05; Figures 2D,E).

### 3.2 Melatonin Attenuated Inflammation and Inflammasome Formation in GIH Offspring

**FIGURE 3:**
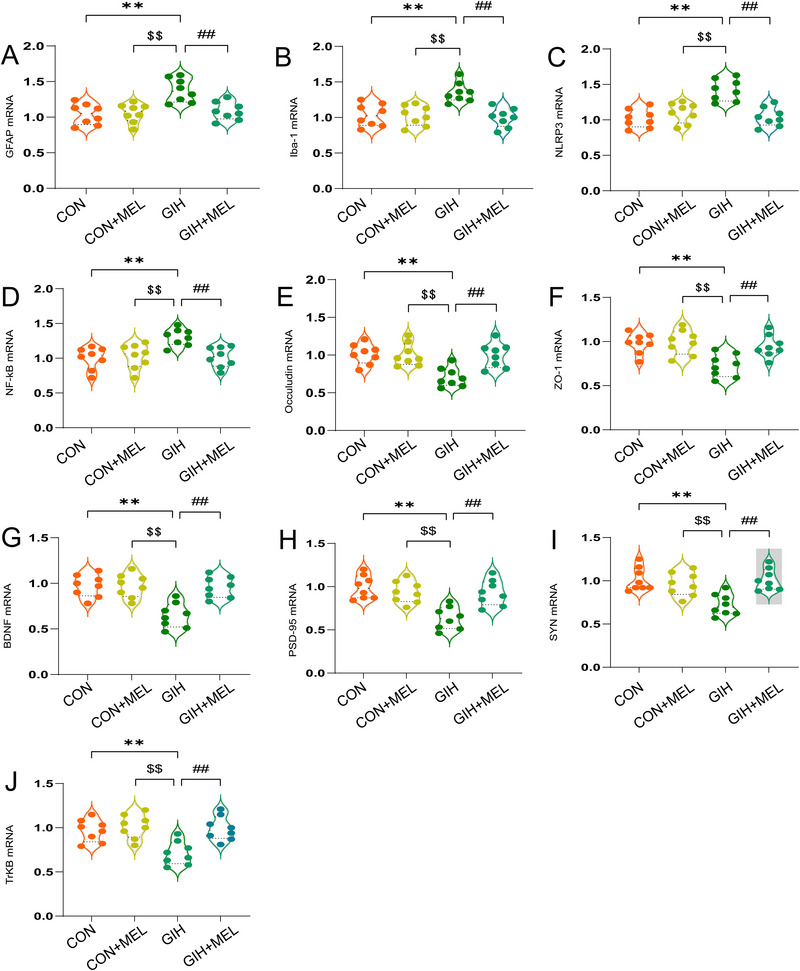
Melatonin attenuated gestational intermittent hypoxia (GIH)‐induced inflammation, glial cell activation, blood‐brain barrier (BBB) leakage, and synaptic dysfunction in offspring mice. qPCR analysis of mRNA levels of (A) GFAP, (B) Iba‐1, (C) NLRP3, (D) NF‐κB p65, (E) occludin, (F) ZO‐1, (G) BDNF, (H) PSD‐95, (I) SYN, and (J) TrkB. All data are expressed as means ± SEM (*n* = 8). *p < 0.05, **p < 0.01 versus the CON group; ^$^ *p* < 0.05, ^$$^ *p* < 0.01 versus the CON+Mel group; ^#^ *p* < 0.05, ^##^ *p* < 0.01 versus the GIH group (one‐way ANOVA with Tukey's post hoc test).

**FIGURE 4:**
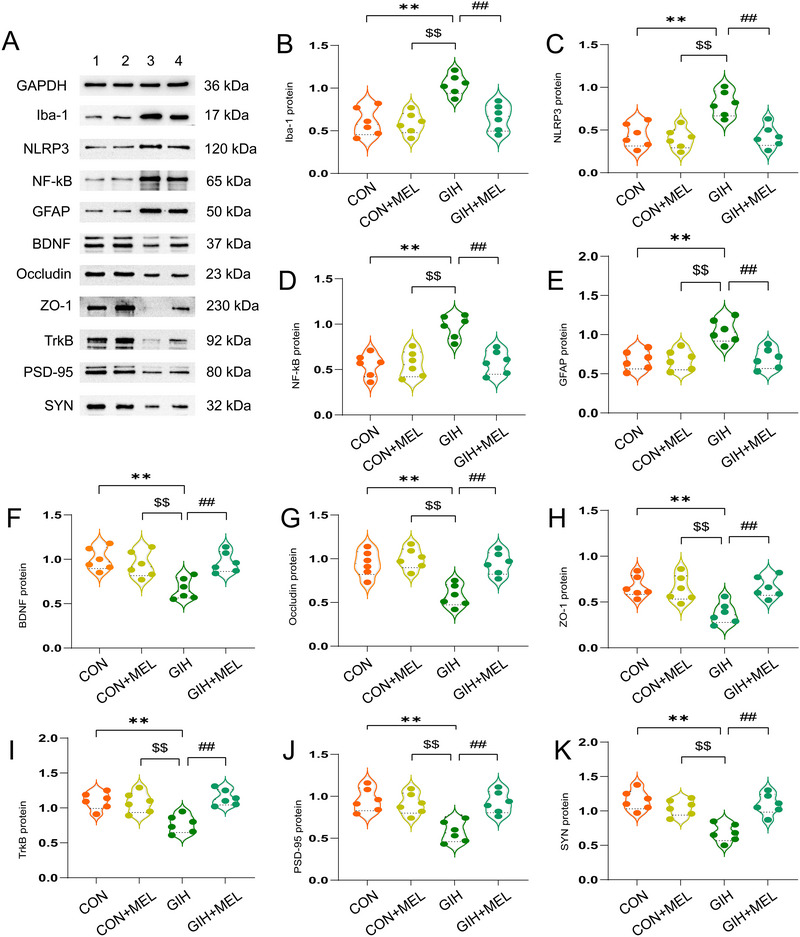
Melatonin suppressed gestational intermittent hypoxia (GIH)‐induced inflammation, glial cell activation, blood‐brain barrier (BBB) leakage, and synaptic dysfunction in offspring mice. (A) Western blot bands. (B–K) Western blot quantification of protein levels: (B) Iba‐1, (C) NLRP3, (D) NF‐κB p65, (E) GFAP, (F) BDNF, (G) occludin, (H) ZO‐1, (I) TrKB, (J) PSD‐95, and (K) SYN. All data are expressed as means ± SEM (*n* = 6). *p < 0.05, **p < 0.01 versus the CON group; ^$^ *p* < 0.05, ^$$^ *p* < 0.01 versus the CON+Mel group; ^#^ *p* < 0.05, ##*p* < 0.01 versus the GIH group (one‐way ANOVA with Tukey's post hoc test).

Notable differences in the mRNA and protein abundances of key inflammatory markers were observed among the groups. Two‐way ANOVA of mRNA expression levels revealed significant group effects for GFAP (*F*_(3,28)_ = 9.964, *p < *0.01), Iba‐1 (*F*_(3,28)_ = 10.99, *p < *0.01), NLRP3 (*F*_(3,28)_ = 14.47, *p < *0.01), and NF‐κB p65 (*F*_(3,28)_ = 7.255, *p < *0.01) (Figures 3A–D). Similarly, significant inter‐group differences were noted in the levels of these proteins (GFAP: *F*_(3,20)_ = 11.09, *p < *0.01; Iba‐1: *F*_(3,20)_ = 13.49, *p < *0.01; NLRP3: *F*_(3,20)_ = 12.32, *p < *0.01; NF‐κB p65: *F*_(3,20)_ = 14.37, *p < *0.01 (Figures 4B–E). Compared with the CON and Mel groups, GIH exposure significantly increased the mRNA and protein levels of GFAP, Iba‐1, NLRP3, and NF‐κB p65 in the hippocampus (all *p < *0.05; Figures 3A–D and 4B–E), indicating that GIH exposure led to the activation of hippocampal glial cells and a corresponding inflammatory response in the brains of the offspring. However, melatonin treatment significantly reduced the GIH‐induced upregulation of GFAP, Iba‐1, NLRP3, and NF‐κB p65 expression at both the mRNA and protein levels (all *p < *0.05; Figures 3A–D and 4B–E). These observations suggest that melatonin attenuates hippocampal inflammation, suppresses inflammasome generation, and dampens glial cell activation in mice exposed to GIH.

### 3.3 Melatonin Protected Mice from GIH‐Induced BBB Leakage

Significant differences in the expression levels of tight junction‐related markers were observed among the groups, at both the mRNA level (occludin: *F*_(3, 28)_ = 8.692, *p < *0.01; ZO‐1: *F*_(3, 28)_ = 7.693, *p < *0.01) (Figures 3E,F) and protein level (occludin: *F*_(3, 20)_ = 13.33, *p < *0.01; ZO‐1: *F*_(3, 20)_ = 7.740, *p < *0.01) (Figures 4G,H). Compared with the CON and Mel groups, GIH exposure significantly decreased both the mRNA and protein levels of occludin and ZO‐1 (all *p < *0.05; Figures 3E,F and 4G,H). Conversely, significant increases in the mRNA and protein levels of these markers were observed in the GIH+Mel group relative to the GIH group (both *p < *0.05; Figures 3E,F, and 4G,H). Collectively, these outcomes demonstrated that GIH induces BBB leakage in offspring mice, while melatonin treatment can mitigate this GIH‐mediated BBB disruption.

### 3.4 Melatonin Modulated Synaptic Protein Synthesis and Release through the BDNF/TrkB Signaling Pathway in GIH‐Exposed Mice

RT‐qPCR and immunoblotting analyses revealed significant inter‐group differences in both the mRNA and protein levels of the cognitive‐related synaptic proteins PSD‐95 and SYP (mRNA: PSD‐95 [*F*_(3, 28)_ = 11.42, *p < *0.01], SYN [*F*_(3, 28)_ = 9.29, *p < *0.01] [Figures 3H,I]; protein: PSD‐95 [*F*_(3, 20)_ = 11.03, *p < *0.01], SYN [*F*_(3, 20)_ = 14.70, *p < *0.01] [Figures 4J,K]). Compared with the CON and Mel groups, GIH exposure significantly reduced both the mRNA and protein levels of PSD‐95 and SYP (all *p < *0.05; Figures 3H,I, and 4J,K). Meanwhile, melatonin treatment (GIH+Mel group) markedly increased the mRNA levels of PSD‐95 and SYP relative to the GIH group (all *p < *0.05; Figures 3H,I). Corresponding increases were observed at the protein level (all *p < *0.001) (Figures 4J,K). Analysis of the BDNF/TrkB signaling pathway revealed significant inter‐group differences at both the mRNA level (BDNF: *F*_(3, 28)_ = 13.00, *p < *0.01; TrkB: *F*_(3, 28)_ = 9.04, *p < *0.01) (Figures 3G,J) and the protein level (BDNF: *F*_(3, 20)_ = 8.59, *p < *0.01; TrkB: *F*_(3, 20)_ = 10.77, *p < *0.01) (Figures 4F,I). Both the mRNA and protein levels of BDNF and TrkB were significantly decreased in the GIH group compared with those in the CON and Mel groups (all *p < *0.05; Figures 3G,J and 4F,I). Additionally, compared with the GIH group, BDNF/TrkB mRNA and protein levels were notably elevated in the GIH+Mel group (all *p < *0.05; Figures 3G,J and 4F,I). This suggested that melatonin attenuated the GIH‐induced damage to hippocampal synaptic proteins in mice, with the BDNF signaling pathway potentially mediating this protective effect.

## 4 Discussion

### 4.1 GIH Exposure Induced Learning and Memory Impairment, Inflammation, Glial Cell Activation, BBB Integrity Disruption, and Synaptic Protein Damage in Offspring Mice

In this study, we found that late gestational hypoxia exerts deleterious effects on cognitive functions, glial cell marker levels, BBB permeability, neuroinflammation, and synaptic protein expression in offspring mice (Table 2). We further observed that GIH elicits inflammatory activity in the pubertal brain, alongside structural and functional damage to BBB tight junctions. Furthermore, these alterations were found to be coupled with a commensurate impairment in memory function and synaptic plasticity. Notably, we demonstrated that melatonin significantly attenuates the GIH‐mediated activation of glial cells and the increase in BBB permeability, thereby protecting against the occurrence of inflammation, synaptic dysfunction, and hippocampal‐dependent cognitive impairments in offspring mice.

**TABLE 2:** Summary of the molecular changes across the experimental groups.

	Iba‐1 protein and mRNA	NLRP3 protein and mRNA	NF‐κB protein and mRNA	GFAP protein and mRNA	BDNF protein and mRNA	Occludin protein and mRNA	ZO‐1 protein and mRNA	TrkB protein and mRNA	PSD‐95 protein and mRNA	SYN protein and mRNA
GIH vs. CON	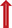	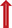	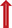	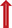	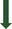	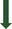	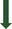	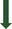	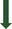	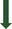
GIH vs. CON + MEL	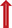	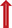	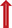	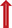	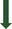	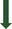	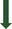	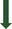	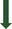	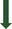
GIH + MEL vs. GIH	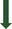	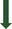	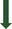	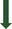	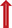	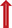	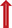	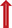	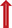	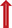

*Note*: ↑ upregulated protein levels (red), ↓ downregulated protein levels (blue).

While we showed that IH during pregnancy results in adverse maternal and neonatal outcomes, the enduring effects on offspring pathophysiology remain insufficiently characterized. Rodent brain development occurs in three stages, with the third stage corresponding to the seventh and eighth months of human gestation (Mabry et al. 2023; Semple et al. 2013). During this period, the cerebral cortex and subcortical regions undergo gradual maturation. In our previous research, we observed that exposure to environmental stressors or inflammation during this developmental window (GD 15–21) can lead to persistent behavioral and cognitive impairments in the offspring. We believe that this stage is uniquely susceptible to hypoxia. Moreover, because the incidence of OSA in humans increases during late pregnancy, we chose to implement IH during this phase (Khalyfa et al. 2017; Guntupalli et al. 2015). This approach potentially enhances experimental sensitivity and more accurately replicates the clinical impact of gestational OSA on offspring development.

In addition to adverse perinatal outcomes (Meers and Nowakowski 2022), hypoxia during pregnancy can also increase the risk of chronic diseases, such as neurodevelopmental disorders, in offspring during later life (Meers and Nowakowski 2022; Gluckman et al. 2008). Studies using animal models have demonstrated that offspring exposed to hypoxia during pregnancy exhibit a range of hypoxia‐sensitive behavioral deficits, including impairments in social and cognitive functions and exacerbations of repetitive and anxiety‐like behaviors (Vanderplow et al. 2022; Wang et al. 2021; Piešová et al. 2020). Recent findings have indicated that late gestational CIH promotes sex‐ and age‐specific changes in pubertal offspring, including in social and memory functions and repetitive behaviors (Mabry et al. 2023). Consistent with previous studies, we found that GIH led to decreases in the percentages of time spent and distance swam in the target quadrant in the MWM test, suggesting that GIH impaired learning and memory abilities in offspring mice.

At present, the main determinants of cognitive impairment in OSA patients include sleep fragmentation and IH, with the latter considered the critical factor driving cognitive decline (Parer 1982; Ducsay et al. 2018). It is hypothesized that maternal IH induced by OSA reduces placental oxygen supply to the fetus. Furthermore, umbilical cord blood analyses indicate that snoring episodes during gestation correlate with heightened fetal erythropoietic activity. These data suggest that the fetus might experience intermittent intrauterine hypoxia secondary to maternal OSA. Clinical studies have shown that OSA‐associated hypoxia may induce gray matter and neuronal damage in multiple cerebral regions, particularly the hippocampus and frontal cortex (Sforza et al. 2004), resulting in diminished attention, reduced processing speed, and impaired executive function in infants and children (Poets 2020). While it is suspected that the mechanism underlying GIH‐induced cognitive impairment in offspring involves hypoxic insult during critical windows of neurodevelopment and synaptogenesis, the specific mechanisms remain largely unexplored.

Under conditions of IH, astrocytes become activated (Yang et al. 2024; Johnson et al. 2018), initiating an inflammatory cascade that leads to the impairment of BBB integrity. This process involves endothelial cell dysfunction and structural disruption of tight junctions and their constituent components, which ultimately increases BBB permeability (Roche et al. 2024). Research has linked OSA to heightened BBB permeability, potentially attributable to neuroinflammation (Kerner and Roose 2016). Studies have shown that procoagulant activity is increased in the serum of patients with OSA. Additionally, circulating plasma exosomes from children with OSA were shown to disrupt barrier integrity in an in vitro BBB model.

The ZO‐1 protein bridges the carboxylic terminus of occludin, junctional adhesion molecules, or actin, thereby functioning as a scaffold for tight junctions and contributing to the maintenance of BBB integrity. Studies have shown that the interaction between occludin and ZO‐1 is essential for the selective permeability barrier function of endothelial cells (Wevers and de Vries 2016). Moreover, the occurrence of ischemia–reperfusion events decreases the expression of these proteins, leading to an abnormal increase in vascular permeability (Sugiyama et al. 2023). While previous work has demonstrated that 15 days of IH exposure enhances BBB permeability and claudin 1 and 12 mRNA expression (Roche et al. 2024), our current data indicate that GIH exposure significantly decreases occludin and ZO‐1 levels while increasing GFAP and Iba‐1 compared to normoxic controls. These findings imply that GIH exposure impairs learning and memory abilities in offspring, characterized by astrocyte activation, the triggering of inflammatory pathways, and compromised BBB integrity.

Recently, it was suggested that astrocyte activation mediated by the NLRP3 inflammasome plays a significant role in neuroinflammatory diseases associated with hypoxia (Wu et al. 2021). Furthermore, inhibiting the activation of the NLRP3 inflammasome has been shown to alleviate neuroinflammation following brain injury (Mao et al. 2017). In clinical research, monocytes from patients with severe OSA exhibit elevated NLRP3 activity, which correlates directly with the apnea‐hypopnea index (AHI) and the severity of hypoxia (Díaz‐García et al. 2022). In animal models, long‐term exposure to IH increases the activity of the NLRP3 inflammasome, while NLRP3 deficiency or inhibition protects the brain from IH‐induced inflammation, oxidative stress, and cell death. While our findings were consistent with the involvement of this pathway, our study uniquely demonstrated that when IH is induced during late gestation, NLRP3 expression remains elevated through to adolescence in the offspring. These observations suggest that neuroinflammation and increased BBB permeability may be related to GIH‐induced cognitive impairment. Consequently, these results indicate that the effects of GIH are not merely transient; rather, they exert a sustained impact on cognitive function through these persistent inflammatory mechanisms.

In rodents, stress during pregnancy leads to enduring epigenetic modifications in the offspring, which encompass persistent alterations in the structure and biological function of microglia. Additionally, prenatal stress is associated with an increase in spontaneous apneic episodes and a diminished ventilatory response to hypoxia in early postnatal rat pups. In our previous research, we found that exposure to inflammation or stress during pregnancy leads to lasting cognitive impairments in offspring, which are associated with changes in the levels of synaptic proteins, including PSD‐95 and SYN (Zhang et al. 2024). Consistent with our previous studies, the current results indicated that GIH similarly leads to a decrease in the mRNA and protein levels of PSD‐95 and SYN in the hippocampus, which are associated with cognitive function in offspring.

Furthermore, we observed that both the mRNA and the protein levels of BDNF and TrkB were reduced in the hippocampus of the GIH group. These data suggest that the downregulation of BDNF and TrkB plays a critical role in the GIH‐induced cognitive impairment. Research indicates that the BDNF/TrkB signaling pathway exerts a crucial regulatory influence on synaptic consolidation and memory function (Saral et al. 2021; Lu et al. 2024). We speculate that the reduction in BDNF levels is linked to the upregulation of neuroinflammatory regulatory pathways. While a recent meta‐analysis identified no significant effect of IH exposure on cerebral BDNF expression, the results suggested that BDNF levels are negatively associated with the duration of the hypoxic phase (El Amine et al. 2024). This finding suggests that extended hypoxic phases are required to achieve a significant decrease in BDNF levels. Nevertheless, our experimental findings indicate that, in comparison with mice whose mothers were subjected to a normoxic environment, those exposed to GIH experienced a notable reduction in BDNF mRNA and protein levels. This discrepancy might be attributed to the duration and mode of hypoxia, as well as the specific developmental stage during which the exposure occurred.

### 4.2 Melatonin Alleviated Cognitive Impairment, Neuroinflammation, Glial Cell Activation, BBB Leakage, and Synaptic Protein Dysfunction in GIH‐Exposed Mice

To date, research relating to the modulation of the damage induced by IH during late gestation remains scarce. Given that late pregnancy represents a critical window for neurodevelopment, it is considered the optimal stage to initiate melatonin intervention (Joseph et al. 2024), including for examining its potential protective effects against GIH exposure when administered during late gestation. In the present study, we found that melatonin treatment significantly attenuated the impairment of learning and memory in the offspring of GIH‐exposed mice. Specifically, the distance swam in the water maze was significantly shorter in the melatonin treatment group than in the GIH‐exposed group, and showed no significant difference compared with the normoxic treatment group. This suggested that melatonin completely offset the neurocognitive damage caused by GIH, although it provided no additional benefits to the control offspring. Similar neuroprotective trends are observed in clinical contexts; for instance, a retrospective study demonstrated that patients with MCI who received daily melatonin performed significantly better on neuropsychological assessments than those without the regimen (Cardinali et al. 2012). Furthermore, animal experiments also revealed that chronic melatonin administration may prevent aging‐associated cognitive decline (Melhuish Beaupre et al. 2021).

Although melatonin has been found to decrease the inflammatory response and prevent cognitive impairment, few studies have examined these effects within the context of GIH. Recent evidence suggests that when mothers exposed to inflammation receive melatonin pre‐treatment, their offspring experience a lower incidence of preterm delivery and perinatal brain injury (Lee et al. 2019). This protective effect may be attributed to the anti‐inflammatory properties of melatonin, which modulates NLRP3 inflammasome function through the regulation of multiple proteins and pathways (Arioz et al. 2021). Specifically, NF‐κB signaling serves as a primary regulator for the priming phase of NLRP3 activation (Hardeland 2018). An animal study revealed that maternal melatonin administration reduces the concentrations of placental proinflammatory cytokines and NF‐κB p65, while also improving offspring neurobehavioral development (Lee et al. 2019). Consistent with these reports, our data showed that melatonin administration reduced the number of glial cells, along with the levels of NF‐κB p65 and NLRP3, compared with those observed in the GIH group. These results support the conclusion that melatonin reduces the GIH‐induced inflammatory response in the offspring hippocampus. Additionally, melatonin demonstrates significant protective effects against cognitive impairment by mitigating inflammation (Thangwong et al. 2023) and BBB damage (Wei et al. 2024). Previous research indicates that melatonin preserves BBB integrity by preventing the loss of tight junction proteins, specifically occludin, claudin‐5, and ZO‐1 (Wei et al. 2024). Consistent with these data, our results showed that melatonin alleviated the increase in BBB permeability and reversed the loss of occludin and ZO‐1 in offspring mice. These findings suggest that melatonin exerts anti‐inflammatory effects and preserves BBB integrity during recurrent gestational hypoxia, thereby safeguarding neural development and cognitive function in offspring.

Melatonin is essential for enhancing cognitive function and mitigating the progression of Alzheimer's disease. It exhibits potent neuroprotective effects by suppressing inflammation and modulating neuronal synaptic plasticity. Several studies have reported that melatonin regulates synaptic plasticity and the expression of synaptic proteins within the brain (Thangwong et al. 2022; Ren et al. 2024; Zhang et al. 2024). Specifically, melatonin has been shown to alleviate the maternal sleep deprivation‐induced decline in SYN and PSD‐95 levels in the hippocampus through the modulation of the BDNF/TrkB signaling pathway (Zhang et al. 2024). However, no prior study has investigated whether melatonin can reduce GIH‐induced synaptic dysfunction in offspring. Our study is the first to demonstrate that melatonin can restore the mRNA and protein levels of SYN, PSD‐95, and components of the BDNF/TrkB pathway relative to controls. Whether the neuroprotective effects observed during adolescence translate into delayed neurodegeneration remains to be determined. Long‐term follow‐up research could help reveal if early melatonin treatment preserves cognitive function in aged C57BL/6J mice, particularly at the age when these mice typically begin to exhibit cognitive decline.

Despite these findings, the precise mechanisms governing the protective effects of melatonin remain unclear. It is generally accepted that melatonin receptors (MT1​ and MT2​) are widely distributed across various brain regions, including the cerebral cortex, hippocampus, and hypothalamus, where they participate in synaptic functions, such as neurotransmitter release. In addition to the classical membrane receptors and the non‐receptor‐mediated antioxidant actions previously described (Slominski et al. 2023), melatonin's cutaneous actions are now understood to entail direct modulation of nuclear receptors. Research suggests that pharmacological concentrations of melatonin and its indolic or kynuric metabolites activate the aryl hydrocarbon receptor (AhR); at higher micromolar levels, these metabolites activate peroxisome proliferator‐activated receptor gamma (PPARγ), providing a receptor‐mediated mechanism for their cytoprotective effects under oxidative stress and supporting their therapeutic use in skin diseases (Slominski et al. 2023). Future studies should prioritize: (i) investigating the receptor‐mediated mechanisms underlying the effects of melatonin and its metabolites in the context of GIH‐induced cognitive impairment; (ii) profiling the epigenetic marks that sustain anti‐inflammatory gene expression; and (iii) translating these findings by correlating maternal melatonin metabolites with neurodevelopmental outcomes in children born to mothers with sleep apnea.

The present study has some limitations. First, while previous data indicate that sex differences exist regarding susceptibility to GIH, the current behavioral and molecular assessments were conducted exclusively in male C57BL/6J mice. Notably, Tai et al. 2011 reported that melatonin (5 mg/kg) alleviates hypoxic–ischemic injury in female Sprague–Dawley rat pups to a greater extent than in males, highlighting the need for sex‐specific studies of GIH and melatonin. Second, cognitive performance was evaluated solely via the MWM test, and anxiety‐like behaviors were not assessed. Finally, we examined only the short‐term benefits of melatonin; the long‐term impacts of treatment following GIH exposure remain unexplored.

## 5 Conclusion

The present study demonstrated that GIH‐exposed mice exhibit cognitive deficits, accompanied by inflammasome formation, neuroglial activation, increased BBB permeability, and synaptic dysfunction. Interestingly, melatonin demonstrated significant protective effects against these GIH‐induced complications. Thus, acting as an anti‐inflammatory mediator, melatonin exerts neuroprotective effects on offspring in the context of gestational sleep‐disordered breathing, with effects that persist into adolescence. Our findings offer new insights into the mechanisms by which GIH induces cognitive dysfunction and highlight the potential of melatonin in mitigating GIH‐related complications.

## Author Contributions

**Xue‐yan Li**: designed the experiments, performed the behavioral tests, and wrote the manuscript. **Yue‐Ming Zhang**: designed the experiments and performed the western blotting and rt‐pcr. **Fei‐Hu**: performed the behavioral tests and analyzed the data, as well as made the figures. **Shi‐Kun Fang**: performed the behavioral tests. **Gui‐Hai Chen**: designed the experiments, funding acquisition, and reviewed and edited the manuscript. **Yu‐Wang**: designed the experiments, funding acquisition, and reviewed and edited the manuscript.

## Funding

The present work received financial support from the National Natural Science Foundation of China (grant no. 81671316) and the Natural Science Research Project of Anhui Educational Commitee (2022AH050759).

## Ethics Statement

All animal procedures were conducted in strict accordance with the humane care guidelines established by the Laboratory Animal Science Association and the Animal Science Center of Anhui Medical University (no. LLSC20190710).

## Conflicts of Interest

There are no conflicts of interest in this study.

## Data Availability

Datasets used and analyzed for the current study can be obtained from the corresponding author upon request.
